# Dynamic Hierarchical Sleep Scheduling for Wireless *Ad-Hoc* Sensor Networks

**DOI:** 10.3390/s90503908

**Published:** 2009-05-25

**Authors:** Chih-Yu Wen, Ying-Chih Chen

**Affiliations:** Department of Electrical Engineering, Graduate Institute of Communication Engineering, National Chung Hsing University, Taichung 402, Taiwan; E-Mail: d9664106@mail.nchu.edu.tw

**Keywords:** wireless sensor networks, scheduling management, sensing coverage

## Abstract

This paper presents two scheduling management schemes for wireless sensor networks, which manage the sensors by utilizing the hierarchical network structure and allocate network resources efficiently. A local criterion is used to simultaneously establish the sensing coverage and connectivity such that dynamic cluster-based sleep scheduling can be achieved. The proposed schemes are simulated and analyzed to abstract the network behaviors in a number of settings. The experimental results show that the proposed algorithms provide efficient network power control and can achieve high scalability in wireless sensor networks.

## 1. Introduction

Recent advances in microelectro-mechanical systems are driving the developments of low-cost and and low-power wireless sensors, with diverse applications in the physical world in areas such as environmental monitoring, disaster recovery, industrial process control, and smart environments. With sensors placed close to an event, wireless sensor networks can observe the phenomenon and receive data. However, having too few active sensors or excessive ones may result in reduced sensing coverage or severe interference, which will have a great influence on network performance features such as energy and bandwidth efficiency, and sensing quality. Therefore, sensing scheduling schemes may be implemented to tackle basic problems of sensor networks (e.g. energy constraints and communication interference) in order to reduce energy consumption and prolong network lifetime.

Sensor scheduling aims to maintain a balance of network resources. Recent research has found that significant energy savings can be achieved by dynamic power management in sensor networks [1-7]. To achieve this sensing process, sensors are scheduled to execute the sensing task. Hence, reducing the sensing redundancy and maintaining sufficient sensing coverage and network connectivity are critical requirements in sensor networks. In addition, the two issues of energy constraint and communication interference have to be considered together with both the network connectivity and data gathering strategy. In this work, two sensor scheduling protocols, *Centralized Adaptive Scheduling Algorithm* (CASA) and *Distributed Adaptive Scheduling Algorithm* (DASA), are proposed to address the application scenario of typical surveillance systems in a cluster-based network topology, where both connectivity and coverage constraint are taken into consideration to achieve performance balance.

For the CASA scheme, given the local information such as neighboring connectivity, the round determination problem may be solved centrally by the clusterheads. For the DASA scheme, as the clusterhead broadcasts a message to start the scheduling assignment, each sensor initializes a random waiting timer with a value which is related to the cluster topology and the neighbor information. If the random waiting timer expires, then the sensor broadcasts a message proclaiming that it is a good candidate to be a group member, which also serves to notify its neighbors that it has a higher priority for the sensing task. Based on the received messages from its neighboring cluster members, each cluster member may use the data gathering strategy (detailed in Section 3.3) to schedule itself to a specific round.

In order to facilitate performance evaluation of a sensor scheduling protocol design, two analytical models, a neural network model and a probabilistic model, are proposed. For the CASA approach, a neural network model is built up to approximate the desire performance. For the DASA approach, a probabilistic model using the concept of geometry is applied to abstract the properties of the algorithm. Moreover, based on the analysis, the sensor lifetime and cluster lifetime is further explored to show how the operations of the proposed schemes may prolong the network lifetime.

The organization of this paper is as follows: Section 2 reviews the current literature on the sensor scheduling management. Section 3 describes the system model and algorithm for sensor scheduling in a cluster-based network topology. In Section 4, a neural network model and a probabilistic model are built up to approximate the desire performance and estimate the sensing rounds of the proposed schemes. Section 5 summarizes the performance of the proposed scheduling methodology. Finally, Section 6 draws conclusions and shows future research directions.

## 2. Literature Review

A large number of sensor scheduling and coverage maintenance protocols have been proposed [8-35]. However, due to the sensing objectives, these management protocols can be different. Yan *et al.* [1] presented an energy-efficient sensing protocol to achieve the desired sensing coverage. Nodes decide their active periods by exchanging reference points among neighbors. In [2], the authors investigated coverage intensity of the proposed sleep scheduling protocols. Ren *et al.* [3] provided a generic analytical framework that can be widely used for sensing scheduling protocol design with detection quality requirements. Turau *et al.* [4] tried to route packet with the minimum time and energy and aimed to distribute the transmission time slots dynamically among sensor nodes such that the network congestion can be avoided.

Hohlt *et al.* [5] proposed a scheduling scheme for considering energy savings in a data collection process. Schrage *et al.* [6] applied an ant colony optimization method for scheduling the visiting order of targeted areas in the sensing field such that their energy consumptions are minimized. Decker *et al.* [7] developed a scheduler to manage the competition for resources among different sensing tasks at a single sensor node. Chamberland *et al.* [8] investigated the relationship between sleeping duration, detection delay and energy consumption in a stationary sensing field. References [9, 10, 11] are clustering-based protocols that attempt to minimize the energy dissipation in sensor networks.

Cheng *et al.* [12] proposed a bio-inspired scheduling scheme which is a kind of adaptive scheduling scheme which uses only local information for making scheduling decisions. Premkumar *et al.* [13] considered the problem of quickest detection of an intrusion using a sensor network, keeping only a minimal number of sensors active. In [14] and [15], randomized scheduling algorithms are proposed for monitoring a field to detect intrusion objects. The authors study the performance of the randomized scheduling algorithm and explore the impact of the size of intrusion object on the sensor network's configuration.

Since energy efficiency and reasonable sensing coverage can be achieved by exploiting the sensing spatial redundancy, redundant sensors may be turned off to save energy [16, 17, 18, 19]. However, the network connectivity is not considered in those schemes. In order to further reduce energy and computational overhead, some scheduling schemes [2, 16, 19, 20] operate without the location information or time synchronization. Although the joint problem of coverage and connectivity is considered in [21, 22, 23, 24, 25], the optimization of the sensing spatial redundancy is not taken into account. A survey of energy-efficient scheduling mechanisms in sensor networks is detailed in [26].

In contrast, the approaches of this paper consider coverage, connectivity, and sensing spatial redundancy simultaneously in order to improve energy efficiency in a hierarchical network structure. For the CASA approach, the clusterhead collects local topology information to manage the sensing schedule centrally. By approximating the network behavior throughout the neural network learning process, the clusterhead may be able to roughly predict the performance of the scheduling management. For the DASA approach, the setting of the random waiting timer allows each sensor to exploit the information about coverage, connectivity, and sensing spatial redundancy such that a balance of network resources can be maintained. Due to the randomized property of the waiting timer, the probabilistic model is proposed to abstract global network behavior. The comparison of the proposed approaches and the other cluster-based schemes [10][11] is further discussed in Section 5.

## 3. Dynamic Sensor Scheduling Algorithms

This section describes two scheduling management schemes for organizing the sensing tasks, the Centralized Adaptive Scheduling Algorithm (CASA) and the Distributed Adaptive Scheduling Algorithm (DASA). The main assumptions of the network are: (1) All sensors are homogeneous with the same transmission range; (2) The sensors are fixed without location information; (3) Symmetric communication channel: all links between sensors are bidirectional; (4) All sensors perform the sensing task periodically. Note that there are no base stations to coordinate or supervise activities among sensors.

### 3.1. Cluster Formation for Scheduling Management

When sensors of a network are first deployed, they may apply the Clustering Algorithm via Waiting Timer (CAWT) from [27] to partition the sensors into clusters. Each sensor sets a random waiting timer, broadcasts its presence via a ‘*Hello*’ signal, and listens for its neighbor's ‘*Hello*.’ The sensors that hear many neighbors are good candidates for initiating new clusters; those with few neighbors should choose to wait. By adjusting randomized waiting timers, the sensors can coordinate themselves into sensible clusters, which can then be used as a basis for further communication and data processing.

Sensors update their neighbor information (i.e. a counter specifying how many neighbors it has detected) and decrease the random waiting time based on each ‘new’ *Hello* message received. This encourages those sensors with many neighbors to become clusterheads. The updating formula for the random waiting time of sensor *i* is:

(1) $$WT_{i}^{\left(k+1\right)}=\gamma \cdot WT_{i}^{\left(k\right)},$$

where $WT_{i}^{\left(k\right)}$ is the waiting time of sensor *i* at time step *k* and 0 < *γ* < 1 is inversely proportional to the number of neighbors. Therefore, if the timer expires, then the sensor declares itself to be a clusterhead, a focal point of a new cluster. However, events may intervene that cause a sensor to shorten or cancel its timer. For example, whenever the sensor detects a new neighbor, it shortens the timer. On the other hand, if a neighbor declares itself to be a clusterhead, the sensor cancels its own timer and joins the neighbor's new cluster.

**Figure 1.**
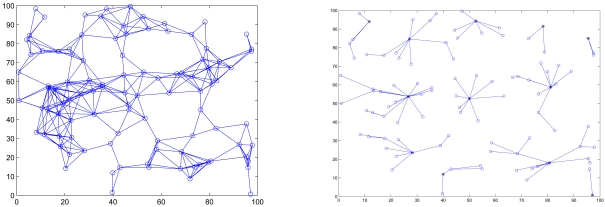
The connectivity of the network (left); clusters are formed in a random network of 100 sensors with *R/l* = 0.17 (right).

After applying the CAWT, there are three different kinds of sensors: (1) the clusterheads (2) sensors with an assigned cluster ID (3) sensors without an assigned cluster ID, which will join any nearby cluster and become 2-hop sensors. In this phase, each sensor initiates two rounds of local flooding to its 1-hop neighboring sensors, one for broadcasting sensor ID and the other for broadcasting cluster ID, to select clusterheads and form 2-hop clusters. Figure 1 shows the network connectivity and cluster formation of a random network of 100 sensors with *R/l* = 0.17, where *R/l* is the ratio of transmitting range *R* to the side length *l* of the square. Thus, the topology of the ad-hoc network is now represented by a hierarchical collection of clusters.

Assume that a cluster of sensor nodes share a common view of a local clock time [28], so that all these nodes can coordinate in the sensor scheduling operation. Given the cluster-based network topology, each cluster member is assumed to sense only once during a sensing cycle *T_cycle_* in a cluster. That is, $T_{\text{cycle}}=\sum_{\ell }^{N_{RG}}T_{RG_{\ell }}$, where *N_RG_* is the number of sensing rounds in a cluster and *T_RG_ℓ__* is the sensing duration of round *ℓ*. For sensor scheduling management, there are three kinds of sensors: (1) sensing nodes: executing the sensing task; (2) relay nodes: maintaining the cluster connectivity for intra-cluster communication; (3) gateway nodes: maintaining the network connectivity for inter-cluster communication. Note that the relay nodes may execute the sensing task in the later round and the gateway nodes may perform the sensing task and the relay transmission during the scheduling operation.

### 3.2. Centralized Adaptive Scheduling Algorithm (CASA)

There are many possible data gathering strategies to accomplish the sensing tasks. In many applications, the monitored area may be used to determine the group members in a specific round. For instance, since the core sensing field is covered by the sensing area of 1-hop cluster members in the cluster-based topology, the round determination problem can be expressed by the coverage subject to the number of 1-hop cluster members covered in the round sensing area,

(2) $$\underset{i\in RG_{\ell }}{\min}\cup O_{i}$$

subject to: *i, j* ∈ *RG_ℓ_*, $j\notin S_{b}^{\left(i\right)}$, ((∪*H*_1_(*i*)+*RG_ℓ_*)∩*H*_1_) = *H*_1_, where *O_i_* represents the coverage overlap of sensor *i* with other round group members, *H*_1_(*i*) is the set of neighboring 1-hop cluster members of sensor *i, RG*_(_*_ℓ_*_)_ is the set of round group members of round *ℓ*, sensors *i* and *j* belong to the set of round group members *RG_ℓ_*, *H*_1_ is the set of 1-hop cluster members, and $S_{b}^{\left(i\right)}$ is the set of neighboring sensors of sensor *i*. The rationale for using the constraint in (2) is to avoid heavy overlap between sensors in the same round. However, without location information, it may be not easy to cover the desired area and minimize the coverage overlap in each round. Hence, the optimization of the coverage may be modified to satisfy the problem constraint by the following scheme.

#### The Scheduling Scheme

When developing the sensing schedule, two rounds of local flooding are initiated in order to gather topology information for the clusterhead in the 2-hop cluster structure. Hence, given the local information such as neighboring connectivity, a clusterhead may choose a 2-hop cluster member and a 1-hop relay node to initialize the proposed scheduling algorithm. After that, the clusterhead may randomly pick 1-hop cluster members, which have no communication links with the chosen group members, as the new round group members. The purpose for this selection policy is to reduce the overlap between group members in the same round. Note that the relay node can be selected as the group member in the following round since it is not responsible for sensing at this round. If all the 2-hop cluster members have been selected for initializing the sensing rounds, the clusterhead will select a 1-hop cluster member for starting the new round. This procedure is repeated until all of the cluster members have been assigned. Then, with a common local clock time in the cluster, the clusterhead triggers two rounds of 1-hop flooding for broadcasting the sensor scheduling information throughout the 2-hop cluster topology.

Observe that the overlap is only approximately minimized; in our experiments we have noticed that the answers tend to be close to the optimal. The pseudo-code of the proposed algorithm is presented in Table 1, where *H*_1_(*m*) is the set of neighboring 1-hop cluster members of sensor *m* in a cluster, *H*_2_(*n*) is the set of neighboring 2-hop cluster members of sensor *n* in a cluster, *RG_ℓ_* is the set of round group members of round *ℓ*, *U* is the set of cluster members, *H*_1_ and $H_{1}^{\prime }$ are the sets of 1-hop nodes, *H*_2_ is the set of 2-hop nodes, and *N_RG_* is the number of sensing rounds in a cluster.

#### Maintenance of Network Connectivity

After establishing the sensing schedule in each cluster, network connectivity may be maintained with two phases of operation (observation and confirmation phases). The period of the observation phase may last several sensing cycles (*nT_cycle_, n* > 1), which allow the sensors to learn about the scheduling operation of their neighboring sensors in nearby clusters. During the observation phase, each sensing node and relay node executes a 1-hop broadcast at the beginning of its active period in the sensing schedule such that the sensing nodes can assign the gateway sensors for inter-cluster communication and data dissemination. The broadcast message includes the sensor node ID and the sensing cycle time *T_cycle_* for the gateway nodes to initialize the next relay transmission.

There are four possible scenarios when determining the gateway nodes: (1) When the sensing node receives only one broadcast message from an active node in the nearby cluster during its sensing period, these two nodes form a pair of distributed gateways. Hence, a sensing node or a relay node in the nearby cluster may be a gateway under this condition; (2) If the sensing node receives multiple broadcast messages from the same nearby cluster, the nearest active node in this specific cluster might be chosen as a gateway node based on distance information, which could be estimated by the received signal strength. Similar to Scenario 1, a sensing node or a relay node may be a gateway in this case; (3) When no broadcast message is received during the sensing period, the sensing node may choose the nearest node in an adjacent cluster as a gateway node; (4) If the clusters are too far apart (outside the range of communication *R*), no gateway sensors will be assigned.

**Figure 2.**
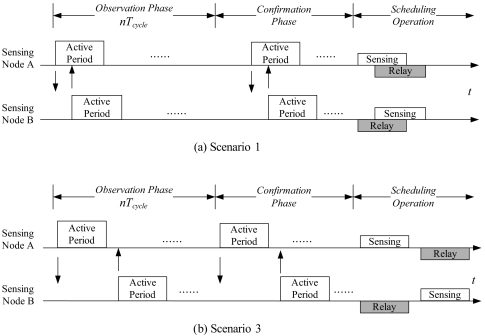
The procedures of selecting a pair of distributed gateways in Scenario 1 (a) and Scenario 3 (b).

Built upon the learning process in the observation phase, the sensing node and the candidate of the gateway node acknowledge the role assignment in the confirmation phase. Thus, each pair of distributed gateways send 1-hop broadcast messages to confirm the gateway selection with each other. Accordingly, the result of gateway selection is that each round group member assigns a single member of each nearby clusters such that network connectivity during the sensor scheduling operation may be assured. Therefore, the CASA approach provides a virtual backbone for sensing coverage and network connectivity maintenance. The procedures of gateway selection is depicted in Figure 2. Figure 2 (a) and Figure 2 (b) describe the operation period of a pair of distributed gateways in Scenarios 1 and 3, respectively. Given the local common clock, the time stamp of the received message, and the duration of the sensing cycle *T_cycle_*, the sensing nodes A and B may work cooperatively as a pair of distributed gateways to adjust their active periods for data dissemination. An example which highlights network coverage and connectivity analysis is further illustrated in Section 5.

### 3.3. Distributed Adaptive Scheduling Algorithm (DASA)

#### The Setting of Waiting Timer

The distributed method operates much like the CAWT [27] in utilizing a random timer. As the clusterhead broadcasts a message to start the scheduling assignment, sensor *i* initializes a random waiting timer with a value $WT_{i}^{\left(0\right)}$:

(3) $$WT_{i}^{\left(0\right)}=\frac{1}{N_{\text{hop}}}\cdot T_{i}\cdot \beta^{N_{b}^{\left(i\right)}},$$

which is related to the cluster topology and the neighbor information. Note that *T_i_* is a sample from the distribution *C*+*λ*·*U*(0, 1), where *C* and *λ* are positive numbers, which are used to specify the sampling range of the waiting time, and *U*(0, 1) is a uniform distribution. *N_hop_* is the number of hops from the clusterhead to the cluster member, $N_{b}^{\left(i\right)}$ is the number of neighboring cluster members of sensor *i, β* is a positive number with 1 < *β*. The rationale for the settings in equation (3) is that, due to the overlap of sensing area in a cluster, the coverage overlap of a 1-hop cluster member is usually larger than that of a 2-hop cluster member. This suggests that a 2-hop cluster member may be a good candidate to initialize a round group. On the other hand, a 1-hop cluster member may choose to wait and join the round group later. Furthermore, a cluster member with more neighbors may have a lower priority to execute the sensing task since its sensing area may be covered by the nearby cluster members.

If the random waiting timer expires (i.e. *WT_i_* = 0), then sensor *i* broadcasts a message proclaiming that it is a good candidate to be a group member, which also serves to notify its neighbors that it has a higher priority for the sensing task. For its neighboring sensor *j*, the update formula of the random waiting timer may be given by:

(4) $$WT_{i}^{\left(k+1\right)}=(1+\alpha )\cdot WT_{i}^{\left(k\right)},$$

where $WT_{i}^{\left(k\right)}$ is the waiting time of sensor *j* at time step *k* and 0 < *α*. The setting of *α* can be attributed to the fact that the neighboring nodes receiving the broadcasting message increase their waiting timers such that they may work in different rounds and the sensing redundancy may be suppressed in each sensing round.

#### The Scheduling Scheme

The message for communication among the cluster members consists of: (1) the ID of the sending sensor, (2) the round ID of the sending sensor, and (3) the relay round ID of the selected relay sensor. At the beginning, the round ID and the relay round ID of each sensor is one and zero, respectively. Based on equation (3), 1-hop cluster members set longer waiting times compared with 2-hop cluster members. When a 2-hop timer expires, the 2-hop cluster member broadcasts a message with the initial round ID 1 and selects its 1-hop parent cluster member as a relay node from the cluster topology. Thus, the selected relay node records its relay round ID and will execute the data dissemination in that sensing round. Since a 1-hop cluster member can report the collected information to the clusterhead directly, relay nodes are not necessary in this case. In order to reduce the overlap of the coverage in each sensing round, the neighboring nodes receiving the broadcasting message update their waiting timers and increase their round IDs by 1 such that they may work in different rounds.

In order to maintain the correct round ID information when receiving multiple messages among neighboring nodes, the ID updating strategy may be described as follows. Given a cluster member with round ID *u* and a message sent by a cluster member with round ID *v*, the cluster member with round ID *u* may update its round ID by *u* = *v* + 1 if the round ID *u* ≤ *v*. Otherwise, the received message is ignored. Accordingly, an update criterion for the sensing node can be derived:

(5) $$\text{Round ID}\;u=\{\begin{array}{cc} v+1, & \text{if}\;u\leq v\\ u, & \text{otherwise}. \end{array}$$

**Figure 3.**
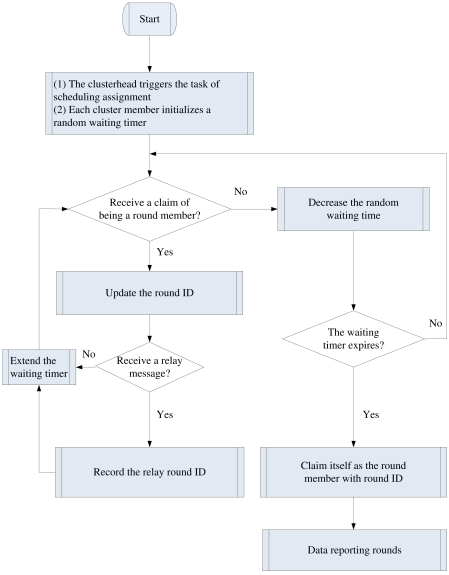
Virtual sensor scheduling flowchart for the DASA algorithm.

By following the above procedures, the round IDs and relay IDs can be determined for each cluster member. Based on the received broadcast messages for updating round ID information from 1-hop cluster members, the clusterhead can obtain the number of sensing rounds *N_RG_* in a sensing cycle. This is because the number of sensing rounds *N_RG_* is equal to the largest round ID of the 1-hop cluster members. Therefore, given a common local clock time in the cluster, the clusterhead may generate two rounds of local flooding for broadcasting the sensor scheduling information throughout the cluster. The procedures of sensor scheduling is outlined in the DASA of Figure 3.

**Figure 4.**
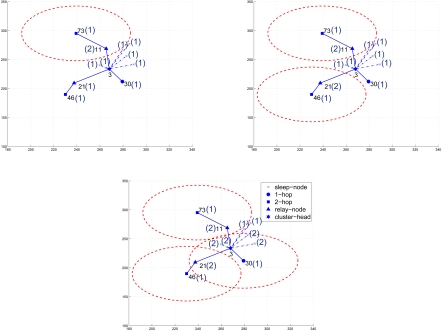
The round ID updating process of the DASA algorithm; the (·) represents the round ID.

Figure 4 illustrates the updating process of round ID among the cluster members for determining the round group members for the first round. At the beginning, sensor 73 broadcasts a message with the initial round ID 1 and selects its 1-hop parent cluster member, sensor 11, as a relay node. Then, sensor 11 records its relay round ID and updates its round ID.

Under the operation of the DASA scheme, pairs of distributed gateways for inter-cluster communication can be decided by applying the same approach as described in Section 3.2. The sensing coverage and connectivity performance will be further explored in Section 5.

## 4. Analysis

Two analytical tools are provided to estimate the number of sensing rounds of the proposed schemes. For the CASA approach, a neural network model is built up to approximate the desire performance. For the DASA approach, a probabilistic model using the concept of geometry and the Lindeberg Theorem [29] are applied to abstract the properties of the algorithm. Moreover, based on the analysis, the sensor lifetime and cluster lifetime is further explored to show how the operations of the proposed schemes may prolong the network lifetime.

### 4.1. Neural Networking for the Centralized Approach

#### Backpropagation Learning Algorithm

This subsection reviews the neural network algorithm [30] for analyzing the performance of the centralized method. Assume that the network under consideration has a general architecture with three layers of neurons. In our case, input and output layer neurons are linear, whereas neurons in the hidden layer are log-sigmoidal. Let the vector pairs in 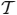 be sample representation of the unknown function *f* : *ℛ^n^* → *ℛ^p^*:

(6) $$T={\left\{\left(X_{k},D_{k}\right)\right\}}_{k=1}^{Q},$$

where *n* is the neuron index range in the input layer, *p* is the neuron index range in the output layer, *X_k_* ∈ *ℛ^n^, D_k_* ∈ *ℛ^p^, Q* is the number of training vector pairs, and *k* is the iteration index. Note that *D_k_* is the desired vector response for the network input *X_k_*. Thus, the mean square error of the entire training set is:

(7) $$\varepsilon =\frac{1}{Q}\sum_{k=1}^{Q}\varepsilon_{k}$$

where $\varepsilon_{k}=\frac{1}{2}{\text{E}}_{k}^{T}E_{k}$, and *E_k_* is the instantaneous error of the training pair (*X_k_, D_k_*). Based on the above description, the update of neuron activations can be formulated as follows.

For the hidden layer:

(8) $${\text{z}}_{h}^{k}=\sum_{i=0}^{n}w_{ih}^{k}\;{\text{x}}_{i}^{k},\;h=1,\ldots ,q$$

(9) $$S\left({\text{z}}_{h}^{k}\right)=\frac{1}{1+e^{-{\text{z}}_{h}^{k}}},\;h=1,\ldots ,q$$

(10) $$w_{ih}^{k+1}=w_{ih}^{k}+\eta (-\frac{\partial \varepsilon_{k}}{\partial w_{ih}^{k}}).$$

For the output layer:

(11) $${\text{y}}_{j}^{k}=\sum_{h=0}^{q}\frac{w_{hj}^{k}}{1+e^{-{\text{z}}_{h}^{k}}},\;j=1,\ldots ,p$$

(12) $$S\left({\text{y}}_{j}^{k}\right)={\text{y}}_{j}^{k},\;j=1,\ldots ,p$$

(13) $$w_{hj}^{k+1}=w_{hj}^{k}+\eta (-\frac{\partial \varepsilon_{k}}{\partial w_{hj}^{k}}).$$

Note that *q* is the neuron index range in the hidden layer, ${\text{x}}_{i}^{k}$ and ${\text{y}}_{j}^{k}$ are the *i*th and *j*th component of the input vector *X_k_* and the output vector *Y_k_*, respectively, $w_{ih}^{k}$ and $w_{hj}^{k}$ are the biases of the hidden and output neurons, respectively, 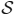
(·) is the signal function, and *η* is the learning rate in the back-propagation algorithm.

#### Estimation of the Number of Sensing Rounds

In order to estimate the number of schedule rounds in a given topology when applying the CASA scheme, the three-layer perceptron neural network is presented. For selecting the network parameters (weights and biases) that best approximate a given function, the backpropagation learning algorithm is considered to minimize the mean square error performance as described in (7).

**Figure 5.**
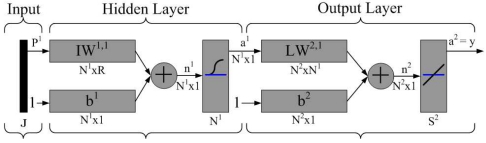
The three-layer perceptron neural network architecture for analyzing the CASA scheme.

Figure 5 illustrates the perceptron network architecture. Note that *J* represents the number of input neurons, which may denote the number of 2-hop cluster members, the number of relay nodes, and the number of 1-hop cluster members. *N*^1^ denotes the number of hidden neurons. In the output layer, *N*^2^ represents the number of neurons, which may denote the network approximation results. Moreover, let IW and LW be the input weight matrix and layer weight matrix for the hidden layer and the output layer, respectively. Let *b*^1^ and *b*^2^ be bias vectors for the hidden layer and the output layer, respectively. Established upon the developed neuron network model, the behavior of the CASA scheme may be abstracted with sensible settings, which is further discussed in Section 5.

### 4.2. Probabilistic Model (PM) for the Distributed Approach

#### Overlap of Geometrical Figures

This subsection introduces a particular problem considering the mean and variance of the overlap of geometrical figures [31]. Given a number of circles placed at random on a plane so that each one may have some or all of its area inside a target area, with reference to the bombing studies, [32] uses the concept of geometry and of probability to explore the fundamentals of this type of problem. The result is represented as the following theorem.

##### Theorem 1

*Let X be a random Lebesgue measurable subset of* n-*dimensional Euclidean space E_n_, with measure μ*(*X*). *For any point x of E_n_, let p*(*x*) = *Pr*(*x* ∈ *X*). *Assuming that the function g*(*x, X*) *is a measurable function of the pair*(*x, X*) *with g*(*x, X*) = 1 *for x* ∈ *X and zero elsewhere, the expected value of the measure* X *is the Lebesgue integral of the function p*(*x*) *over E_n_*.

Suppose that *A* and *C* are the interior of the closed curves. Let the subset *X* be the part of a region *A* in *E*_2_ which is covered by *z C*'s dropped independently and randomly. Denote a reference point *Q* as the centra of the area *C* and assume that there is a frequency distribution *ϕ*(*x, y*) of the position (*x, y*) of *Q*. Based on the above assumptions, now we consider the moments of the area *Y* = *A* − *X* (i.e. the area of *A* not covered by the *z C*'s).

Referring to Theorem 1, the probability of a point (*x*_1_, *y*_1_) in *Y* not being covered by a *C* is:

(14) $$q(x_{1},y_{1})=\int_{T-\bar{C}(x_{1},y_{1})}\int \phi (x,y)\text{dxdy},$$

where *T* − *C̅*(*x*_1_, *y*_1_) is the part of *T* exterior to the area *C*. Therefore, the first moment of *Y*, in the case of *z C*'s, yields:

(15) $$\mu_{Y}^{\left(1\right)}=\int_{A}\int q^{z}(x_{1},y_{1})dx_{1}dy_{1}.$$

similarly, for the *m*th moment, the probability that the points (*x*_1_, *y*_1_), (*x*_2_, *y*_2_), …, (*x_m_, y_m_*) are not covered by a *C* is:

(16) $$q(x_{1},y_{1},x_{2},y_{2},\ldots ,x_{m},y_{m})=\int_{T-{\bar{C}}_{1}-{\bar{C}}_{2}-\ldots -{\bar{C}}_{m}}\int \phi (x,y)\text{dxdy},$$

where *T* − *C̅*_1_ − *C̅*_2_ − *…* − *C̅_m_* is the area of *T* outside *C*'s centered at (*x*_1_, *y*_1_), (*x*_2_, *y*_2_), …, (*x_m_, y_m_*). Thus, the *m*th moment in the case of *z C*'s is given by:

(17) $$\mu_{Y}^{\left(m\right)}=\int_{A}\int \cdots \int_{A}\int q^{z}\left(x_{1},y_{1},x_{2},y_{2},\ldots ,x_{m},y_{m}\right)dx_{1}dy_{1}dx_{2}dy_{2}\ldots dx_{m}dy_{m}.$$

Accordingly, in our case we may interpret *T* as the sensing field in a cluster, let *A* be the sensing field of a given sensor, let *X* be the area covered by its neighboring cluster members, and *Y* will represent the sensing area of a given sensor not covered by its neighboring cluster members. Denote the parameters *z* and *m* as the number of unconnected cluster members and the neighboring cluster members, respectively. Therefore, the *m*th moment $\mu_{Y}^{\left(m\right)}$ may describe the fraction of *A* of a given sensor not covered by its *m* neighboring cluster members.

#### Lindeberg Theorem

This subsection reviews the probability that is used when analyzing the performance of the model. Readers may refer [29] for a complete discussion and proof of the theorem.

Suppose for each *n*

(18) $$\begin{array}{c} \left(X_{11},X_{12},\ldots ,X_{1r_{1}}\right)\\ \left(X_{21},X_{22},\ldots ,X_{2r_{2}}\right)\\ \vdots \\ \left(X_{n1},X_{n2},\ldots ,X_{nr_{n}}\right) \end{array}$$

are independent random vectors. The probability space may change with *n* and (18) is called a *Triangular Array* of random variables. Put *S_n_* = *X_n_*1 + ⋯ + *X_nr_n__*. In the network application, let *X_ni_* be *X_i_* and let *X_i_* take the values 1 and 0 with probability *p_i_* and *q_i_* = 1 − *p_i_*. We may interpret *X_i_* as an indicator that sensor *i* is chosen to be a round member with probability *p_i_* and *S_n_* is the number of members in a round.

Denote *Y_i_* = *X_i_* − *p_i_*. Hence:

(19) $${\text{S}}_{n}^{Y}\equiv \sum_{i=1}^{n}Y_{i}=\sum_{i=1}^{n}X_{i}-\sum_{i=1}^{n}p_{i}=S_{n}-\sum_{i=1}^{n}p_{i},$$

(20) $$E\left[Y_{i}\right]=E\left[X_{i}\right]-p_{i}=0,$$

(21) $$\sigma_{Y_{i}}^{2}=\sigma_{X_{i}}^{2}=p_{i}\left(1-p_{i}\right),$$

(22) $$\sigma_{s_{n}}^{2}=\sum_{i=1}^{n}\sigma_{Y_{i}}^{2}=\sum_{i=1}^{n}\sigma_{X_{i}}^{2}=\sum_{i=1}^{n}p_{i}\left(1-p_{i}\right).$$

For our case, the Lindeberg condition [29] reduces to:

(23) $$\underset{n\to \infty }{\lim}\sum_{i=1}^{n}\frac{1}{s_{n}^{2}}\int_{\left|Y_{i}\right|\geq \varepsilon s_{n}}Y_{i}^{2}dP\leq \underset{n\to \infty }{\lim}\sum_{i=1}^{n}\frac{1}{s_{n}^{2}}\int_{\left|Y_{i}\right|\geq \varepsilon s_{n}}dP=0,$$

which holds because all the random variables are bounded by 1 and [|*Y_i_*| ≥ ∈*s_n_*] → 0 as *n* → ∞.

##### Theorem 2

*Suppose that Y_i_ is an independent sequence of random variables and satisfies E*[*Y_i_*] = 0, $\sigma_{Y_{i}}^{2}=E\left[Y_{i}^{2}\right]$, ${\text{S}}_{n}^{Y}=\sum_{i=1}^{n}Y_{i}$, *and* $s_{n}^{2}=\sum_{i=1}^{n}\sigma_{Y_{i}}^{2}$. *If the Lindeberg condition (23) holds, then* ${\text{S}}_{n}^{Y}/s_{n}\to N\left(0,1\right)$.

Observe that *p_i_* may be described by the overlap fraction of sensor *i* since the sensors with less overlap between its neighboring sensors has a larger chance to be selected as a round group member in the round competition, which coincides with the operation of the DASA and the setting of the waiting timer.

#### Estimation of the Number of Sensing Rounds

Assume that each sensor will be grouped with probability $p_{i}^{\left(k\right)}$ at iteration *k*. Denote the collection of cluster members for selecting the round members at iteration *k* by *V_k_*. Since the round members are selected and removed at each iteration, the collection of sensors at the next iteration, *V_k_*+1, is simply a new and smaller cluster. Thus, by Theorem 2, the distribution of the number of round members at iteration *k* can be approximated by $N\left(\mu_{k},\sigma_{k}^{2}\right)$ with $\mu_{k}=\sum_{i=1}^{m_{k}}p_{i}^{\left(k\right)}$ and $\sigma_{k}^{2}=\sum_{i=1}^{m_{k}}p_{i}^{\left(k\right)}\left(1-p_{i}^{\left(k\right)}\right)$. Once the procedure terminates, the number of iterations is an estimate of the number of rounds formed in the cluster. A statement of procedures for analyzing the DASA is given in Table 2.

### 4.3. Sensor Lifetime and Cluster Lifetime

The main objective of the dynamic sleep scheduling approaches is to extend the lifetime of the clusters so that the network may remain functional longer. Say that the cluster lifetime ends when the first sensor in the cluster fails. Therefore, it is worthwhile to understand the lifetime of individual sensors.

Depending on the traffic model of the network, the expected sensor lifetime may be different. Suppose that the sensors measure periodically and transmit the data back to the clusterhead for further processing with a steady traffic. We also assume that the clusterhead collects the information from cluster members and communicates with the base station with a steady traffic flow [33]. Thus, the expected lifetime $E\left[\Delta T_{i}^{\left(j\right)}\right]$ of sensor *i* at round *j* in a sensing cycle is:

$$E\left[\Delta T_{i}^{\left(j\right)}\right]=p_{i}^{\left(j\right)}\cdot \left(\frac{E_{i}^{\left(j\right)}-E_{i}^{\left(j+1\right)}}{P_{i}}\right),$$

where $p_{i}^{\left(j\right)}$ is the probability of sensor *i* to be a round member at round *j, P_i_* is the power dissipation of sensor *i*, and $E_{i}^{\left(j\right)}-E_{i}^{\left(j+1\right)}$ is the energy consumption at round *j*. Hence, for sensor *i*, the expected energy consumption in a sensing cycle is

(24) $$E_{i}^{\left(sc\right)}=\sum_{j}P_{i}\cdot E\left[\Delta T_{i}^{\left(j\right)}\right]$$

and the expected sensor life time of sensor *i* for being a round group member is given by:

(25) $$E_{RG}\left[T_{i}\right]=\frac{E_{i}^{\left(0\right)}}{E_{i}^{\left(sc\right)}}\cdot T_{\text{cycle}}$$

(26) $$=\frac{E_{i}^{\left(0\right)}}{\sum_{j}P_{i}\cdot E\left[\Delta T_{i}^{\left(j\right)}\right]}\cdot T_{\text{cycle}},$$

where $E_{i}^{\left(0\right)}$ is the initial energy of sensor *i* and $E\left[\Delta T_{i}^{\left(j\right)}\right]$ is the expected lifetime of sensor *i* at round *j* in a sensing cycle *T_cycle_*.

Accordingly, the impact of the sleep scheduling approach on cluster lifetime is further examined. For a cluster without sleep scheduling strategy, the expected lifetime of sensor *i* is:

(27) $${\tilde{E}}_{WS}\left[T_{i}\right]=\frac{E_{i}^{\left(0\right)}}{P_{i}}.$$

For a cluster with sleep scheduling strategy, the expected lifetime of sensor *i* is:

(28) $$E_{S}\left[T_{i}\right]=p_{i}^{\left(ch\right)}\cdot \frac{E_{i}^{\left(0\right)}}{P_{i}}+\left(1-p_{i}^{\left(ch\right)}\right)\cdot E_{RG}\left[T_{i}\right]$$

(29) $$=p_{i}^{\left(ch\right)}\cdot \frac{E_{i}^{\left(0\right)}}{P_{i}}+\left(1-p_{i}^{\left(ch\right)}\right)\cdot \frac{E_{i}^{\left(0\right)}}{\sum_{j}P_{i}\cdot E\left[\Delta T_{i}^{\left(j\right)}\right]}\cdot T_{\text{cycle}},$$

where $p_{i}^{\left(ch\right)}$ is the probability for sensor *i* to be a clusterhead, which may be related to the operation of the clustering algorithm.

Based upon the definition of the cluster lifetime, the cluster lifetime is equal to the minimum of the expected lifetime of sensors. That is, *L̃_ch_* ≡ min*_i_*{*Ẽ_WS_*[*T_i_*]} and *L_ch_* ≡ min*_i_*{*E_S_*[*T_i_*]}. To quantitatively measure how well the cluster lifetime are extended, we introduce a parameter, cluster lifetime factor (CLF). The CLF is defined as the ratio of the cluster lifetime with sleep scheduling and to the cluster lifetime without sleep scheduling. Thus, the CLF is:

(30) $$\text{CLF}\equiv \frac{L_{ch}}{{\tilde{L}}_{ch}}=\frac{\min_{i}\left\{E_{S}\left[T_{i}\right]\right\}}{\min_{i}\left\{{\tilde{E}}_{WS}\left[T_{i}\right]\right\}}.$$

Now we provide an example on how the cluster lifetime can be extended by applying the dynamic scheduling techniques. Assume that sensors of the network have identical initial energy levels and power dissipation. Therefore, the cluster lifetime factor (CLF) yields:

(31) $$\text{CLF}=\min_{i}\left\{p_{i}^{\left(ch\right)}+\left(1-p_{i}^{\left(ch\right)}\right)\cdot \frac{T_{\text{cycle}}}{\sum_{j}E\left[\Delta T_{i}^{\left(j\right)}\right]}\right\},$$

which shows that *CLF* ≥ 1 since the sensing cycle $T_{\text{cycle}}\geq \sum_{j}E\left[\Delta T_{i}^{\left(j\right)}\right]$. That means the cluster can last longer by using sleep scheduling schemes, further extending the lifetime of the network.

### 4.4. Complexity Analysis

This subsection assesses the performance of the proposed schemes in terms of communication and time complexity for network operations.

#### CASA Scheme

When developing the sensing schedule, two rounds of local flooding are initiated in order to gather topology information for the clusterhead in the 2-hop cluster structure. Hence, the time complexity is 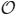
(2) rounds. Next, the clusterhead triggers two rounds of 1-hop flooding for broadcasting the sensor scheduling information throughout the cluster. At last, two rounds of 1-hop flooding is performed for determining the gateway nodes. Thus, the time complexity is 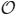
(6) rounds.

Consider a sensor, say sensor *i*, is a clusterhead. Suppose that the total power requirements include the power required to transmit messages *E_T_* and the power required to receive *E_R_*. Therefore, the total energy consumption, *E_sch_*, for scheduling management in the cluster is *E_sch_* = *N_T_* · *E_T_* + *N_R_* ·*E_R_* with:

(32) $$N_{T}=1+4\cdot N_{C}+N_{i},$$

(33) $$N_{R}=N_{i}+4\cdot \sum_{j=1}^{N_{C}}N_{j}+\sum_{k\in S_{b}^{\left(i\right)}}N_{k},$$

where *N_C_* is the number of sensors in the cluster, *N_i_* is the number of the neighboring sensors of sensor *i*, and $S_{b}^{\left(i\right)}$ is the index set of neighboring sensors of sensor *i*. Given the energy consumption analysis above, the communication complexity due to establishing the sensing schedule is 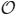
(*N_C_*). Moreover, when operating the sensing task, the energy consumption is related to the number of active sensors in a round. Therefore, the communication complexity for collecting sensing data is 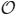
(*M_RG_*), where *M_RG_* is the number of round members in the cluster.

Since the algorithm is mainly executed in the clusterhead, the computation cost analysis of a clusterhead is presented. Based on the procedures of the CASA scheduling scheme in Table 1, it includes the operations needed to check the status of the cluster members and to select round group members. Hence, the computation complexity for scheduling management in a clusterhead is $O\left(N_{C}^{2}\right)$, where *N_C_* is the number of sensors in the cluster.

In order to show the frequency of operations on the system resource and explore the impact of gathering all the information by the clusterhead, memory usage analysis is provided in terms of information processing perspective. Assume each node has a *L*-byte data packet to transmit. Based on the operation of the CASA scheme, the memory usage *M_CASA_* is given by:

(34) $$M_{\text{CASA}}=M_{T}+M_{S}+M_{G}$$

(35) $$=(\sum_{j=1}^{NC}N_{j}+N_{C}+\left|H_{1}\right|+\sum_{k\in H_{i}^{\left(2\right)}}N_{k}+2N_{G})\cdot L,$$

where *M_T_* is the memory usage for gathering topology information, *M_S_* is the memory usage for broadcasting the scheduling information, and *M_G_* is the memory usage for gateway selection, *N_j_* is the number of the neighboring sensors of sensor *j, N_C_* is the number of sensors in the cluster, *H*_1_ is the set of 1-hop nodes, $H_{i}^{\left(2\right)}$ denotes the index set of 1-hop cluster members of cluster *i* with neighboring 2-hop cluster members, and *N_G_* is the number of gateway sensors for inter-cluster communication in the cluster.

By using the CASA algorithm, the periodic on-off scheduling problem can be solved efficiently due to a sleeping schedule for each sensor node in a cluster. However, the drawback is using a centralized accumulator host to gather topology information of each sensor such that it can execute the scheduling management. The problem arises when some of the sensors can not transmit the required information to the accumulator host or the accumulator host malfunctions.

#### DASA Scheme

In the DASA approach, the clusterhead triggers two rounds of 1-hop flooding to initialize the sensor scheduling management process in the 2-hop network topology. Next, 1 round of local flooding is applied for updating the round ID of each node. Then, the clusterhead generates another two rounds of local flooding for broadcasting the sensor scheduling information. Finally, the gateway nodes are selected using two rounds of 1-hop flooding. Therefore, the time complexity is 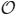
(7) rounds.

Accordingly, the total energy consumption is *E_sch_* = *N_T_* · *E_T_* + *N_R_* · *E_R_*, where:

(36) $$N_{T}=2\cdot \left(1+\left|H_{i}^{\left(2\right)}\right|\right)+3\cdot N_{C},$$

(37) $$N_{R}=2\cdot (N_{i}+\sum_{j\in H_{i}^{\left(2\right)}}N_{j})+3\cdot \sum_{j=1}^{N_{C}}N_{j},$$

where $H_{i}^{\left(2\right)}$ denotes the index set of 1-hop cluster members of cluster *i* with neighboring 2-hop cluster members. Hence, the communication complexity due to scheduling management is 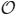
(*N_C_*), *N_C_* is the number of sensors in the cluster. Similar to the CASA scheme, when operating the sensing task, the energy consumption is related to the number of active sensors. Therefore, the communication complexity for collecting sensing data is 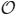
(*M_RG_*), where *M_RG_* is the number of members in each round in the cluster.

Due to the operation of the DASA scheme, the computation burden is distributed among the sensors. Thus, the computation cost analysis is considered with respect to the clusterhead and cluster members, respectively. For the clusterhead, it arranges the sensing schedule based on the largest received round ID. Hence, the computation complexity for updating the round ID in a clusterhead is 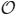
(*N_C_*). For the cluster members, they need to initialize a waiting time, to check if a claim of being a round member is received, to update the round ID, to extend the waiting time, and to check if the waiting timer expires. Therefore, the computation complexity for scheduling management in a cluster member is $O\left(N_{b}^{\left(j\right)}\right)$, where $N_{b}^{\left(j\right)}$ is the number of the neighboring cluster members of sensor *j*.

Suppose that each sensor node has a *L*-byte data packet to transmit. According to the operation of the DASA scheme, the memory usage *M_DASA_* yields:

(38) $$M_{\text{DASA}}=M_{I}+M_{R}+M_{S}+M_{G}$$

(39) $$=(2\cdot \sum_{j=1}^{N_{C}}N_{j}+2\left|H_{1}\right|+\sum_{k\in H_{i}^{\left(2\right)}}N_{k}+2N_{G})\cdot L,$$

where *M_I_* is the memory usage for initializing the procedure of scheduling management, *M_R_* is the memory usage for updating the round ID, *M_S_* is the memory usage for broadcasting the scheduling information, and *M_G_* is the memory usage for gateway selection, *N_C_* is the number of sensors in the cluster, *H*_1_ is the set of 1-hop nodes, and *N_G_* is the number of gateway sensors for inter-cluster communication in the cluster.

Note that although the DASA scheme has a higher time complexity due to the round ID updating process, the DASA allows the cluster members to organize themselves into round groups and complete the scheduling assignment automatically with only local neighboring information.

## 5. Experimental Results

Assume that *N_s_* sensors are uniformly distributed over a square region in two-dimensional space. Parameters for the random waiting timer, number of sensors, and ratio of transmitting range *R* to the side length *l* of the square, *R/l*, are investigated to provide a simulation-based study of the proposed schemes. for the DASA scheme, the parameters (detailed in Section 3.3) for the experiments are given by *C* = 100, *λ* = 10, *α* = 0.5, and *β* = 1.5.

**Figure 6.**
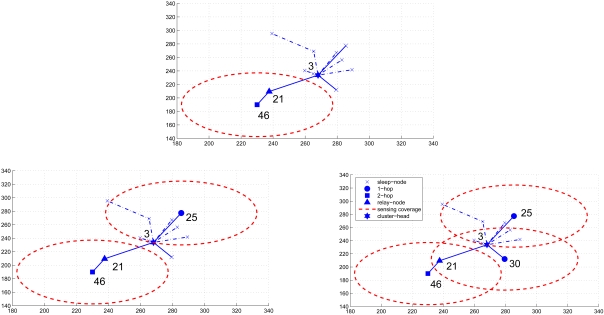
An example of generating a sensing round with the CASA approach.

The first set of experiments illustrates two examples of generating a sensing round with the CASA approach and the DASA approach, respectively. According to the procedures of the CASA approach, as shown in Figure 6, the clusterhead selects a 2-hop cluster member and a relay node, say sensors 46 and 21, to initiate the round generation. For approximately minimizing the overlap of sensing coverage, sensors having no connectivity with sensor 46 may be selected as the round group member. By following the data gathering strategy in Section 3.2, sensors 46, 25, and 30 are chosen to form a sensing round.

**Figure 7.**
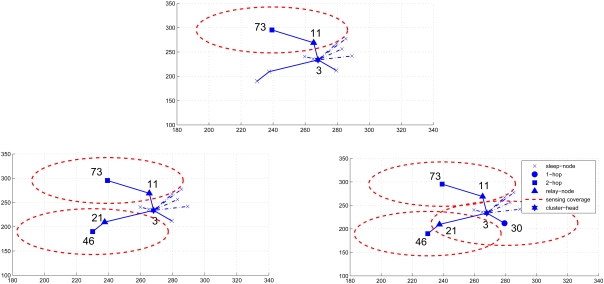
An example of generating a sensing round with the DASA approach.

In Figure 7, based on the settings of the DASA approach, a 2-hop cluster member with a shorter random waiting timer, say sensor 73, broadcasts a message to its neighbors and a 1-hop cluster member, say sensor 11, broadcasts a message to claim its being a relay node for sensor 73. When receiving the broadcasting messages, the neighboring sensors extend their waiting times to reduce the sensing area redundancy and further prepare for being the round group members in the following sensing rounds. Thus, with the data gathering strategy as described in Section 3.3, sensors 73, 46, and 30 form a sensing round. Observe that, as shown in Figures 6 and 7, the proposed approaches avoid heavy sensing redundancy and maintain sufficient sensing coverage.

**Figure 8.**
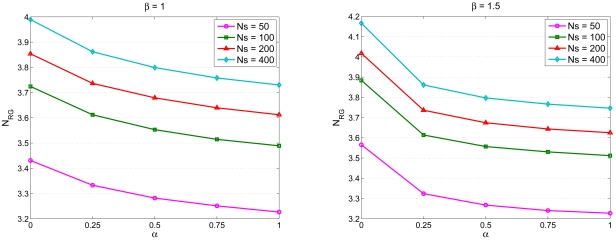
The number of sensing rounds *N_RG_* versus *α* with *β* =1 (left) and *N_RG_* versus *α* with *β* = 1.5 (right).

Given a cluster topology, the second set of experiment studies the impact of parameter settings on network performance. With varying the number of sensors *N_S_*, Figure 8 shows the number of sensing rounds *N_RG_* versus *α* for *β* =*1* (left) and *N_RG_* versus *α* for *β* = 1.5 (right). Given a value of the parameter *β*, the number of sensing rounds *N_RG_* decreases with increasing value of the parameter *α*, which implies that the operation of updating the waiting time when receiving the round ID broadcast from the neighboring sensors may rearrange the sensing schedule and decrease *N_RG_*.

**Figure 9.**
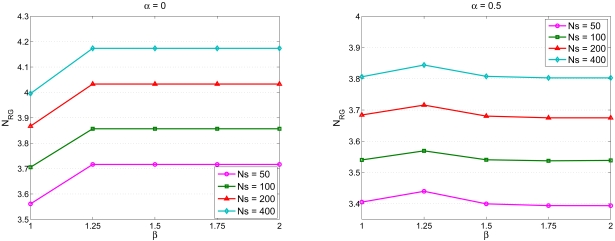
The number of sensing rounds *N_RG_* versus *β* with *α* = 0 (left) and *N_RG_* versus *β* with *α* = 0.5 (right).

Similarly, Figure 9 depicts the number of sensing rounds *N_RG_* versus *β* with *α* = 0 (left) and *N_RG_* versus *β* with *α* = 0.5 (right). Given *α* = 0 (i.e. without applying the update formula), the number of sensing rounds *N_RG_* increases slightly with increasing value of the parameter *β*. This is because the setting of the parameter *β* may allow nearby sensors to work on different rounds such that the sensing redundancy may be suppressed. On the other hand, by applying the update formula (e.g. *α* = 0.5), the parameter *α* may be a dominant factor affecting the number of sensing rounds *N_RG_* since this mechanism allows the sensors to observe the behaviors of their nearby sensors and make adjustments in their waiting times. Therefore, the update operation of the waiting time described in (4) may play a critical role in scheduling management.

**Figure 10.**
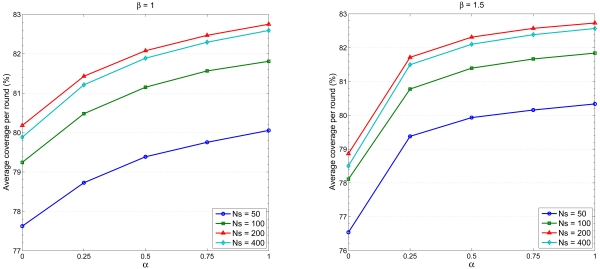
Average coverage per round versus *α* with *β* = 1.0 (left) and with *β* = 1.5 (right).

**Figure 11.**
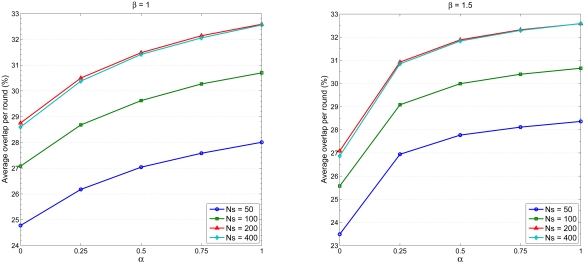
Average sensing overlap per round versus *α* with *β* = 1.0 (left) and with *β* = 1.5 (right).

Furthermore, in order to describe the interaction between the parameter settings and the network performance, Figures 10 and 11 illustrate the average coverage and average sensing overlap per round versus *α* with *β* = 1.0 (left) and with *β* = 1.5 (right), respectively. Observe that in Figure 10, given *β* the average coverage per round increases with increasing value of *α*. As shown in Figure 8, the number of sensing rounds *N_RG_* decreases with increasing value of the parameter *α*, which means the number of active nodes in a round *N_RG_* increases with increasing value of *α* and more sensing overlap may be introduced under this condition (Figure 11). Based on the above results, the parameters *α* = 0.5 and *β* = 1.5 may be sensible settings for balancing the relationship between scheduling management and network performance. Therefore, depending on the requirement of the sensing task, these key parameters may be chosen to achieve desired performance.

**Figure 12.**
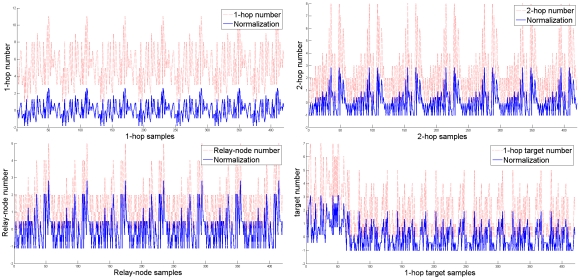
The preprocessing results of the network inputs and targets.

The third set of experiments explores the performance of the neural network model. The efficiency of neural network training can be improved with certain preprocessing steps performing on the network inputs and targets [34]. Figure 12 illustrates the preprocessing results of the network inputs and targets, which transform inputs and targets into a better form and then reverse transformed outputs back to the characteristics of the original target data.

**Figure 13.**
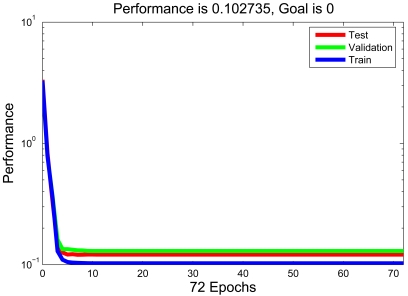
An independent test of network generalization.

**Figure 14.**
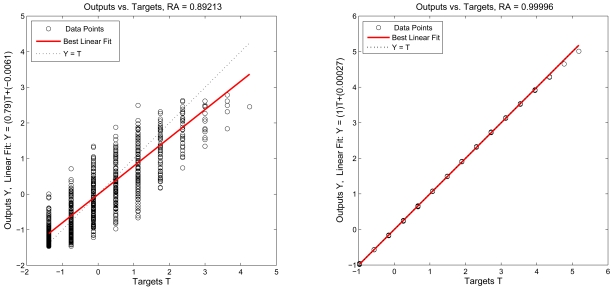
The regression analysis between the network response and the corresponding targets: 1-hop regression analysis (left) and 2-hop regression analysis (right).

Figures 13 and 14 depict the learning and regression analysis of the network. Figure 13 shows that the network is learning since the mean squared error of the network is decreasing to a smaller value. The 6,514 input and target vectors are randomly divided into three sets. Four thousand vectors are used to train the network. Of these vectors 1,257 are used to validate how well the network generalized. Finally, the last 1,257 vectors provide an independent test of network generalization to data that the network has never seen.

Moreover, regression analysis is employed as post-training analysis between the network response and the corresponding targets and three parameters are returned to evaluate the performance. The first two parameters, slope and y-intercept of the best linear regression relate targets to network outputs. If the outputs exactly equal to targets, the slope and y-intercept would be 1 and 0, respectively. For the 1-hop case, slope = 0.79 and y-intercept = −6.1 · 10^−3^. For the 2-hop case, slope = 1.0 and y-intercept = 2.7 · 10^−4^. The third parameter is correlation coefficient between the outputs and targets. When the correlation coefficient is equal to 1, then there is perfect correlation between targets and outputs. In this study, the correlation coefficients of 1-hop regression analysis and 2-hop regression analysis are *RA* = 0.90 and *RA* = 1.00 as shown in Figure 14 (left) and Figure 14 (right), respectively, which therefore illustrate a good fit.

**Figure 15.**
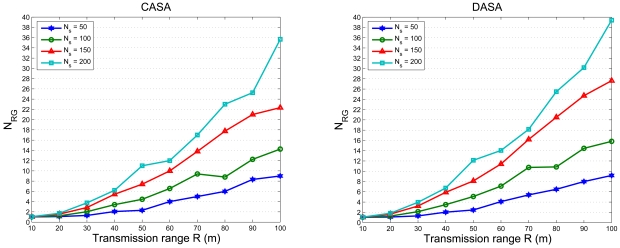
The relationship between the number of sensing rounds *N_RG_* in a cluster and transmission range *R* with varying the number of sensors.

**Figure 16.**
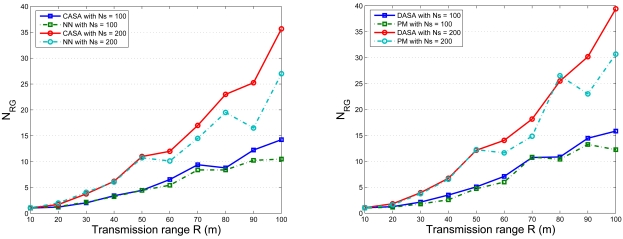
The comparison of the average number of round groups *N_RG_* applying the proposed scheduling schemes and those using the analytical models.

**Figure 17.**
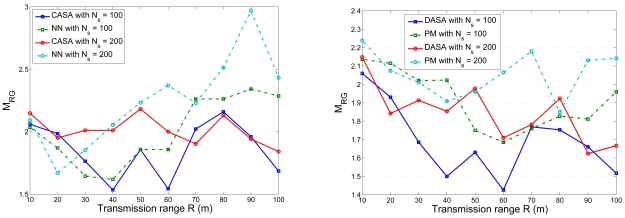
The number of active nodes in a round *M_RG_* with varying the transmission range *R*.

In order to simultaneously consider energy conservation, network connectivity, and the data gathering strategy, the fourth set of experiments investigates the impact of the transmission range *R* on the average number of round groups *N_RG_* in the scheduling operation with *l* = 300 m. Figure 15 shows the relationship between the average number of round groups *N_RG_* and the *R/l* ratio with varying the number of sensors. Figure 16 shows the comparison of the average number of round groups *N_RG_* applying the proposed scheduling schemes and those using the analytical models. To further explore the sensing load in a round, Figure 17 depicts the number of active nodes *M_RG_* in a round with varying the transmission range *R*. The result shows that the average number of group members in a round is between 1.5 and 2.5 for the proposed scheduling schemes.

Observe that, as shown in Figure 15, the average number of sensing rounds *N_RG_* increases as the ratio *R/l* increases (i.e. the transmission power increases). Since larger transmission power allows larger radio coverage, a cluster has more cluster members, which increases the coverage overlap and results in a larger *N_RG_* in a cluster. Thus, a large *R* may result in heavy sensing redundancy due to a large number of neighboring sensors. On the other hand, a small *R* may produce many isolated sensors in a network. Therefore, a sensible transmission range is essential to explore the performance of the scheduling approach. In [35], the authors suggest that $R\approx l\sqrt{\frac{\log l}{N_{s}}}$ may be a good choice for the initial range assignment for sensors in the *2*-dimensional space, where *N_s_* is the number of sensors. As a result, the clusterhead may adaptively manage the scheduling operation based on the data gathering strategy and an appropriate transmission range *R* in order to avoid severe communication interference.

**Figure 18.**
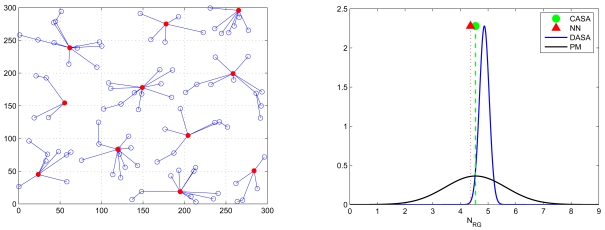
The distribution of the number of sensing rounds *N_RG_* applying the proposed scheduling schemes and those using the analytical models with *N_s_* = 100.

**Figure 19.**
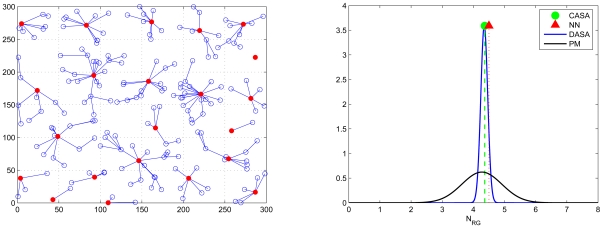
The distribution of the number of sensing rounds *N_RG_* applying the proposed scheduling schemes and those using the analytical models with *N_s_* = 200.

Accordingly, an appropriate transmission range *R* is applied when comparing the proposed schemes and the analytical models. Figures 18 and 19 show the standard deviation of the mean number of sensing rounds *N_RG_* with *l* = 300 m, *N_s_* = 100, and *R* ≈ 50 m and with *l* = 300 m, *N_s_* = 200, and *R* ≈ 33.387 m, respectively. In Figure 18 (right), the neural network (NN) architecture well approximates the CASA performance since the NN retains global information from the training process. Because of the sensor spatial distribution in Figure 18 (left), for the DASA scheme, the result provides evidence that the Probabilistic Model (PM) provides a way to roughly predict the performance of the DASA. However, due to the uniform convergence of the sensor spatial distribution (*N_s_* = 200) in Figure 19 (left), the PM method has done well to describe the performance of the DASA. Moreover, with an appropriate transmission range (with *l* = 300 m, *N_s_* = 100, and *R* ≈ 50 m and with *l* = 300 m, *N_s_* = 200, and *R* ≈ 33.387 m), Figures 18 and 19 show that both the proposed schemes and the analytical models are close, which coincides with the results in Figure 16. Therefore, the average number of round groups *N_RG_* in a sensing cycle can be clearly specified for a sensing task in a cluster.

**Figure 20.**
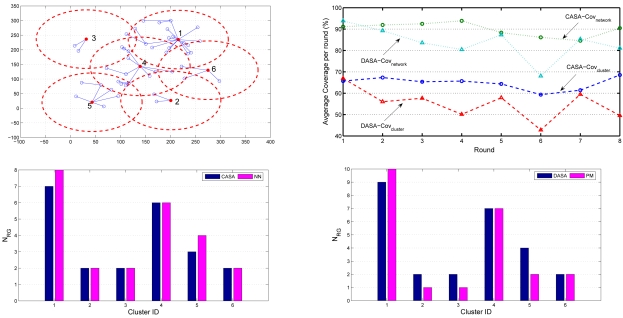
The coverage percentage of the whole network and each cluster (the top-right quadrant); the accuracy of the neural network architecture (the bottom-left quadrant) and the accuracy of the Probabilistic Model (PM) in a random network (the bottom-right quadrant).

Note that Figures 15 and 16 investigate the average number of sensing rounds *N_RG_* considering all clusters in a network. In order to further explore the performance of the proposed schemes and the analytical models, the fifth set of experiments examines the accuracy of the neural network architecture and the accuracy of the Probabilistic Model (PM) in each individual cluster. Given a cluster-based network topology (Figure 20 (top left)), the bottom-left and bottom-right quadrants in Figure 20 show that the accuracy of the network for new data and the approximation of the PM model match the performance of the proposed schemes well, which may provide clusterheads a way to estimate the number of sensing rounds given local topology information. Furthermore, the top-right quadrant in Figure 20 illustrates the coverage percentage of the whole network and each cluster, respectively, which suggests that the proposed schemes allow the network to obtain high coverage percentage with an appropriate sensing range. Notice that the operation of the CASA scheme achieves 65% average sensing coverage in a cluster and 90% average sensing coverage in a random network. This is attributed to the fact that the sensing compensation from nearby clusters results in a higher percentage of sensing coverage in a network. Similarly, given the same network topology, the operation of the DASA scheme achieves 55% average sensing coverage in a cluster and 80% average sensing coverage in a random network. Since the CASA scheme executes the scheduling management in a centralized way, it may have better spatial arrangement of sensors in each round compared with the DASA scheme.

**Figure 21.**
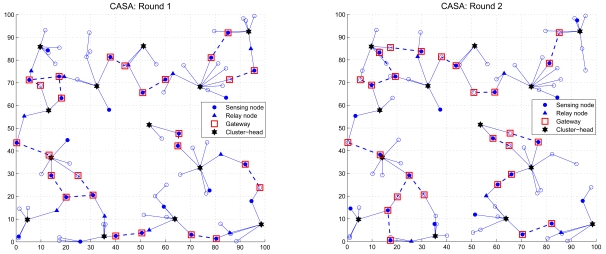
The network connectivity using the CASA scheme in round 1 (left) and round 2 (right).

**Figure 22.**
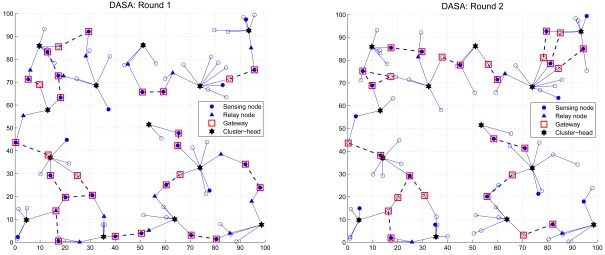
The network connectivity using the DASA scheme in round 1 (left) and round 2 (right).

The sixth set of experiments studies the network connectivity when using the proposed scheduling approaches. Given a random network with *N_s_* = 100 and the period of the observation phase 5*T_cycle_*, Figures 21 and 22 show the maintenance of network connectivity in round 1 and round 2. The circle (‘○’) represents the sleep node and a connection between a pair of distributed gateways (‘□’) is indicated by a dashed line. Observe that in order to conserve energy, the active nodes (the sensing node ‘●’ and the relay node ‘▲’) are good candidates for being gateways in each round. As demonstrated in Figures 21 and 22, although the network may not be fully connected in each round, the operations of the proposed schemes maintain sufficient network connectivity and provide a way for inter-cluster communication and data dissemination.

**Figure 23.**
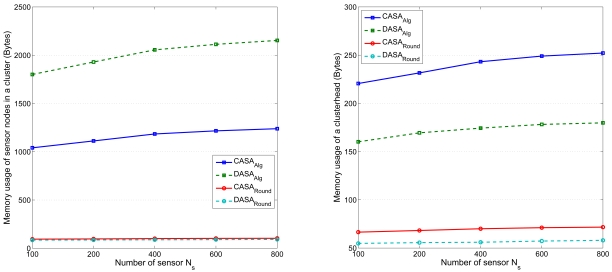
Memory usage of sensor nodes in a cluster versus number of sensors *N_S_* (left); memory usage of a clusterhead versus number of sensors *N_S_* (right).

By following the analysis as detailed in Section 4.4, the seventh set of experiments illustrates the mean memory usage in the cluster members and the clusterhead, respectively. Assume each node has a 36-byte data packet to transmit. Figure 23 (right) depicts the mean memory utilization for gathering the information in a clusterhead. Figure 23 (left) shows the total mean memory usage for running the proposed scheduling algorithms in the sensors in a cluster. As shown in Figure 23 (left), compared with the CASA, the cluster members consume more memory as establishing the sensing schedule with the DASA scheme. The main reason can be contributed to the tracking operation of round ID, which is used to create the order of sensing round. Moreover, for the memory usage in a clusterhead [Figure 23 (right)], due to the centralized operation, the clusterhead using the CASA scheme consumes more memory, compared with the clusterhead using the DASA with only local information. However, when measuring the memory utilization in a sensing round, the memory usage performance of the proposed approaches are comparable since the number of active nodes in a round *M_RG_* is considered to be close in each approach as demonstrated in Figure 17. Accordingly, the current wireless sensor networking products (e.g. Crossbow's IRIS Mote with 8K bytes RAM and 512K bytes Flash or Tmote Sky with 10K bytes RAM and 48K bytes Flash) are capable of running the proposed scheduling schemes.

**Figure 24.**
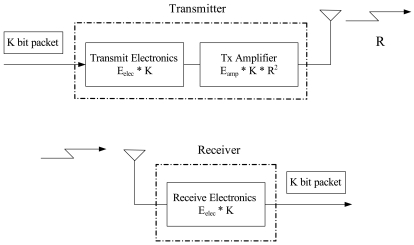
First order radio model as described in [10].

The last set of experiments depicts the energy consumption of the proposed algorithms and compare the results with those of other cluster-based scheduling protocols. Assume that clusters are formed in a random network of 100 sensors with side length *l* = 100 m. A simple model [10] where the radio dissipates *E_elec_* = 50 nJ/bit to run the transmitter or receiver circuitry and *E_amp_* = 100 pJ/bit/m^2^ for the transmit amplifier is applied in order to achieve an acceptable SNR. Suppose an *r*^2^ energy loss due to channel transmission. Thus, to transmit a *K*-bit message a distance *R* using the radio model, the radio expends: *E_T_* = *E_elec_* · *K* + *E_amp_* · *K* · *R*^2^ and to receive this message, the radio expends: *E_R_* = *E_elec_* · *K* (Figure 24). For data dissemination, the cluster-based hierarchical routing protocol [36] may be used for inter-cluster routing. The intra-cluster routing is built upon the node-level topology of cluster, which is obtained by the CAWT mechanism. Thus, data traffic between two clusters will be relayed through the gateway nodes.

**Figure 25.**
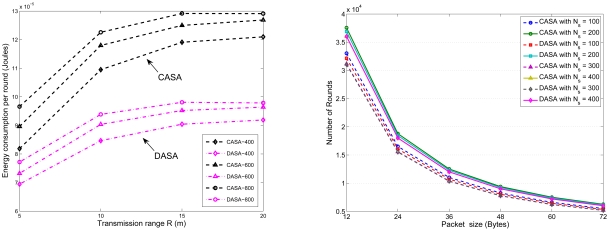
The relationship between energy consumption per round (Joules) and transmission range *R* (left); the number of sensing rounds with varying the size of data packet (right).

Assuming that each node has a 36-byte data packet to transmit, Figure 25 (left) illustrates the average energy consumption per round under different transmission range with varying the number of sensors in the network. Furthermore, with varying the size of data packet in each sensor node, Figure 25 (right) presents the number of sensing rounds given the energy constraint. As expected, due to an increasing packet size and a higher energy dissipation rate, the number of sensing rounds decreases given a fixed initial energy (0.5 J). On the other hand, with an appropriate transmission ranges according to the network density (with *l* = 100 m, *N_s_* = 100, and *R* ≈ 14.14 m; with *l* = 100 m, *N_s_* = 200, and *R* ≈ 10.0 m; with *l* = 100 m, *N_s_* = 300, and *R* ≈ 8.17 m; with *l* = 100 m, *N_s_* = 400, and *R* ≈ 7.07 m) and varying the packet size, the number of sensing rounds in the network with different network densities suffers only small variations, which suggests that the proposed schemes may achieve high scalability for sensor scheduling.

**Figure 26.**
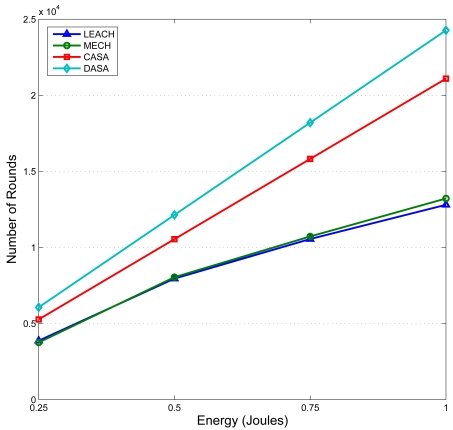
The comparison of the number of rounds as the first sensor node dies in the network using the LEACH, MECH (10 members), CASA, and DASA.

Figure 26 shows the number of rounds when the first node dies in the network using the LEACH [10], the MECH [11], the CASA, and the DASA with varying the initial energy of each node from 0.25 J to 1.0 J. The LEACH and the MECH are clustering-based protocols that tries to minimize the energy dissipation in sensor networks. Observe that the proposed approaches are superior to the LEACH and the MECH approaches, while the number of sensing rounds grows nearly linearly as the initial energy increases. The simulation results demonstrate that the proposed schemes are more energy efficient than the LEACH and the MECH schemes.

## 6. Conclusions

This paper presents hierarchical scheduling algorithms, which use a local criteria to simultaneously undertake the sensing coverage and connectivity such that dynamic cluster-based sleep scheduling can be achieved. An analytical network architecture and a probabilistic model are derived to describe the behaviors of the proposed schemes. The clusterheads may apply the established models to estimate the number of sensing rounds given local topology information. The main objective of the proposed dynamic sleep scheduling approaches is to extend the lifetime of the clusters so that the network may remain functional longer. The experimental results show that the proposed algorithms provide efficient network power control and achieve high scalability in wireless sensor networks.

There are several ways this work may be generalized. For instance, the CASA scheme may exploit the relationship between the monitored area in a cluster and the cluster topology to determine a proper number of group members in a round for the sensing task. Also, the DASA scheme can be generalized to a *d*-hop cluster-based network topology. By following the procedures of the DASA scheme, a *d*-hop cluster member may be a good candidate to initialize a round group and a nearby (*d*-1)-hop cluster member may choose to wait and join the following round group later. The random timer may be adjusted using local information and energy constraints and adapt based on the requirements of the sensing task in order to achieve network robustness and scalability.

In the proposed scheduling solutions, trade-offs are found between model complexity, energy consumption, computational complexity, and sensible model description in real systems. Future plans will involve generalizing the methods to design energy-efficient data dissemination protocols, to consider certain failure scenarios, to explore the sensitivity of the proposed schemes to data gathering strategies and network operation, and to perform actual measurements to investigate the impact of parameter settings on network performance.

## Figures and Tables

**Table 1.** The CASA Scheduling Scheme

Assign *N_RG_* = 0, *ℓ* = 1;
**while** (*U* ≠ *ϕ*) **do**
{
$H_{1}^{\prime }=H_{1}$;
*RG_ℓ_* = *ϕ*;
\* Selecting 2-hop round members *\
**if** (*H*_2_ ≠ *ϕ*)
{
*i* = argmax*_k_*|*H*_1_(*k*)|, ∀ *k* ∈ *H*_2_;
*RG_ℓ_* = {*i*};
$H_{1}^{\prime }=H_{1}^{\prime }-H_{1}(i)$;
*H*_2_ = *H*_2_ − *i*;
}
\* Selecting 1-hop round members *\
**while**$\left(H_{1}^{\prime }\neq \phi \right)$**do**
{
Pick sensor *m*, $m\in H_{1}^{\prime }$;
*RG_ℓ_* = *RG_ℓ_* ∪ *m*;
$H_{1}^{\prime }=H_{1}^{\prime }-H_{1}(m)$;
*H*_1_ = *H*_1_ − *m*;
}
*U* = *U* − *RG_ℓ_*;
*ℓ* = *ℓ* + 1;
*N_RG_* = *N_RG_* + 1;
}

**Table 2.** Procedures of the PM model for analyzing the DASA.

Let *n* be the number of cluster members. *r_k_* is the number of sensors to be removed and *m_k_* is the number of sensors remaining at iteration *k* Assign the probability $p_{i}^{\left(k\right)}$ to sensor *i*, proportional to the fraction not covered by its neighboring cluster members. That is, $p_{i}^{\left(k\right)}\propto Y/A$, as described in Theorem 1. Assign *k* = 0, *m*_0_ = *n, r*_0_ = 0. **while** (*m_k_* − *r_k_*) > 0 $r_{k}=\left\lceil \sum_{i=1}^{m_{k}}p_{i}^{\left(k\right)}\right\rceil$ as detailed in Theorem 2, *m_k_*_+1_ = *m_k_* − *r_k_*, form members of this round, update $p_{i}^{\left(k\right)}$, *k* = *k* +1. **end**
* ⌈·⌉ is the ceiling function.
